# Ex vivo fecal fermentation of human ileal fluid collected after raspberry consumption modifies (poly)phenolics and modulates genoprotective effects in colonic epithelial cells

**DOI:** 10.1016/j.redox.2021.101862

**Published:** 2021-01-12

**Authors:** Sara Dobani, Cheryl Latimer, Gordon J. McDougall, J. William Allwood, Gema Pereira-Caro, José Manuel Moreno-Rojas, Nigel G. Ternan, L. Kirsty Pourshahidi, Roger Lawther, Kieran M. Tuohy, Daniele Del Rio, Gloria O'Connor, Ian Rowland, Tahani Mazyad Almutairi, Alan Crozier, Chris I.R. Gill

**Affiliations:** a Nutrition Innovation Centre for Food and Health, University of Ulster, Coleraine, Northern Ireland, UK; b Environmental and Biochemical Sciences Department, The James Hutton Institute, Invergowrie, Dundee, Scotland, UK; c Department of Food Science and Health, IFAPA-Alameda Del Obispo, SN, Córdoba, Spain; d Altnagelvin Area Hospital, Londonderry, Northern Ireland, UK; e Food Quality and Nutrition Department, Fondazione Edmund Mach, San Michele All'Adige, Italy; f Department of Veterinary Science, University of Parma, Parma, Italy; g Department of Food and Nutritional Sciences, University of Reading, Reading, UK; h Department of Chemistry, King Saud University, Riyadh, Saudi Arabia; i Department of Nutrition University of California, Davis, CA, USA

**Keywords:** Ileostomy, Gastrointestinal microbiota, Raspberry (poly)phenols, Phenolic catabolites, Fecal fermentation, DNA damage, Colon cancer, ANOVA, analysis of variance, ARE, antioxidant responsive element, CRC, colorectal cancer, FBS, fetal bovine serum, GC-MS, gas chromatography-mass spectrometry, GI, gastrointestinal, HO-1, heme oxygenase-1, HPRT, hypoxanthine phosphoribosyl transferase, UHPLC-HR-MS, ultra high-performance liquid chromatography-high-resolution mass spectrometry, n.d., not detected, NQO1, NAD(P)H dehydrogenase quinone-1, Nrf2, nuclear factor (erythroid-derived 2)-like 2, PBS, phosphate-buffered saline

## Abstract

Diets rich in fruit and vegetables are associated with a decreased incidence of colorectal cancer (CRC) due, in part, to the bioactive (poly)phenolic components and their microbiota-mediated metabolites. This study investigated how such compounds, derived from ingested raspberries in the gastrointestinal tract, may exert protective effects by reducing DNA damage. Ileal fluids collected pre- and post-consumption of 300 g of raspberries by ileostomists (n = 11) were subjected to 24 h *ex vivo* fermentation with fecal inoculum to simulate interaction with colonic microbiota. The impact of fermentation on (poly)phenolics in ileal fluid was determined and the bioactivity of ileal fluids pre- and post fermentation investigated. (Poly)phenolic compounds including sanguiin H-6, sanguiin H-10 and cyanidin-3-*O*-sophoroside decreased significantly during fermentation while, in contrast, microbial catabolites, including 3-(3′-hydroxyphenyl)propanoic acid, 3-hydroxybenzoic acid and benzoic acid increased significantly. The post-raspberry ileal fermentate from 9 of the 11 ileostomates significantly decreased DNA damage (~30%) in the CCD 841 CoN normal cell line using an oxidative challenge COMET assay. The raspberry ileal fermentates also modulated gene expression of the nuclear factor 2–antioxidant responsive element (Nrf2-ARE) pathway involved in oxidative stress cytoprotection, namely Nrf2, NAD(P)H dehydrogenase, quinone-1 and heme oxygenase-1. Four of the phenolic catabolites were assessed individually, each significantly reducing DNA damage from an oxidative challenge over a physiologically relevant 10–100 μM range. They also induced a differential pattern of expression of key genes in the Nrf2-ARE pathway in CCD 841 CoN cells. The study indicates that the colon-available raspberry (poly)phenols and their microbial-derived catabolites may play a role in protection against CRC *in vivo*.

## Highlights

• Health effects of dietary (poly)phenols linked to interactions within the GI tract.
• Ileostomy-based bioavailability studies allow effective interrogation of the GI tract.
• Fecal fermentation of raspberry-enriched ileal fluid, increases phenolic content.
• Raspberry ileal fluid fermentates & phenolic acids reduce DNA damage in colonocytes.
• Cytoprotective Nrf2-ARE pathway modulated by ileal fluid fermentates & phenolic acids.

## Graphical abstract

**Figure:**
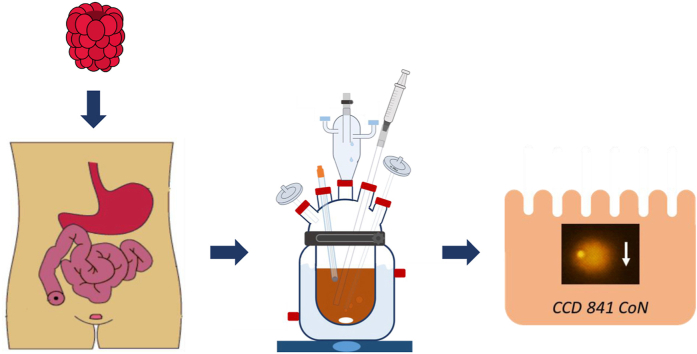

## 1 Introduction

(Poly)phenols contribute to chronic disease risk-reduction associated with diets rich in fruit and vegetables [1,2] through their pleiotropic bioactivity including anti-inflammatory, anti-proliferative and anti-genotoxic activities [[3], [4], [5]]. The bioavailability of many (poly)phenol classes has traditionally been deemed low [6], but if the contribution of their gut microbiota-mediated catabolites is considered, there is a growing realisation it is much greater than classically reported [[7], [8], [9], [10], [11]].

The putative health benefits of dietary (poly)phenols probably occur via interactions within the gastrointestinal (GI) tract, including the production of bioactive microbiota-derived catabolites [[10], [11], [12], [13]], maintenance of a beneficial microbiota population, and protection against epithelial damage associated with GI disease [14,15]. Consequently, numerous studies have emulated effects on the GI tract using a variety of *in vitro* models [16] including INFOGEST which advocates a standardised model including buccal simulation [17]. These models demonstrate that (poly)phenols have different stabilities and are likely available in amounts that could modulate physiological processes. They compare potential in-gut availability but cannot comprehensively mimic the dynamic and active processes associated with the digestion of food when consumed by humans [18]. However, ileostomy-based bioavailability studies provide a unique insight into events taking place in the GI tract, facilitating identification of compounds which, in individuals with an intact colon, would pass from the small to the large intestine and ultimately exert their effects [[19], [20], [21], [22], [23], [24], [25]].

While numerous studies have reported bioactivities for (poly)phenols, especially anti-genotoxicity effects, many are of limited physiological relevance as they largely ignored the importance of the digestive process on the composition, structure and bioactivity of the compounds in the GI tract [[26], [27], [28], [29], [30], [31]]. Mechanistically, Nuclear factor erythroid 2-related factor 2 (Nrf2) likely mediates anti-genotoxic activity as it is modulated, directly or indirectly, by many (poly)phenols [32]. Moreover, Nrf2 is a target for the prevention of carcinogenesis [33,34] as it regulates the expression of a range of intracellular endogenous antioxidant and phase II detoxifying enzymes including; NAD(P)H:quinone oxidoreductase 1 (NQO1), heme oxygenase 1 (HO-1), thereby providing cytoprotection. This function is exemplified by the Nrf2 knockout mouse model (Nrf2KO: APC^min/+^ x Nrf2^−/−^) which exhibits a reduced response to oxidative stress and enhanced tumorigenesis [35].

In the current study an *ex vivo* approach coupling *in vivo* digestion by ileostomates to *ex vivo* fecal fermentation was used to produce physiologically relevant samples to explore whether (poly)phenols derived from consumption of raspberries could beneficially impact gut health, in terms of reduction of DNA damage and Nrf2-ARE pathway modulation.

## 2 Methods

### 2.1 Chemicals and reagents

Minimum essential media, penicillin/streptomycin, sodium pyruvate, non-essential amino acid, and foetal bovine serum were purchased from Gibco Life Technologies Ltd (Paisley, Scotland, UK). HPLC-MS grade methanol and acetonitrile were acquired from Panreac (Barcelona, Spain) and formic acid (HPLC-MS grade) from Sigma-Aldrich (Madrid, Spain). Cyanidin-3-*O*-glucoside and pelargonidin-3-*O*-glucoside were obtained from Apin Chemicals (Abingdon, Oxford, U.K.), cyanidin-3-*O*-sambubioside-5-*O*-glucoside was supplied by (Poly)phenols (Sandnes, Norway), and punicalagin was obtained from Chromadex (U.S.A). All other chemicals were purchased from Sigma-Aldrich (Poole, Dorset, UK).

Note, the names for phenolic catabolites used in this paper is based on the nomenclature thesaurus of Kay et al. [36] and the nomenclature used in cited papers has, as necessary, been adjusted.

### 2.2 Ileostomy feeding study

The ileal fluid samples were obtained from an earlier raspberry feeding study [23] approved by the Ethics Committee at the University of Ulster (Ref No. 11/NI/0112) and carried out in accordance with The Code of Ethics of the World Medical Association (Declaration of Helsinki) for experiments involving humans. Briefly, after a 48 h diet low in (poly)phenols and an overnight fast, 11 healthy ileostomy volunteers provided a baseline ileal fluid sample. They then consumed 300 g of homogenised raspberries and a second ileal fluid sample was collected 8 h post-consumption. Samples were processed as described previously [23], and stored at −80 °C.

### 2.3 Ex vivo fermentation of ileal fluid

To simulate the colonic environment ileal fluid samples were fermented with a human fecal inoculum for 24 h in a pH, temperature-controlled *in vitro* batch culture model. In brief, sterile water-jacket vessels were filled aseptically with 85 mL of basal nutrient media. The vessels were equipped with a continuous oxygen-free nitrogen supply and an automated pH-regulation system connected with NaCl (0.5 M) and HCl (0.5 M) reservoirs. A single fecal donor, fulfilling the pre-requisites described previously [37], provided verbal consent for the fecal matter to be used for the experiments in compliance with the ethics procedures of the University of Reading. A single donor was used to minimise variation as a result of metabotype [38]. Once weekly, for 4 weeks, the donor provided a stool sample, which was collected and within an hour homogenised via a stomacher (Seward, Norfolk, UK) and diluted 1:10 using sterile pre-reduced phosphate-buffered saline (PBS). A 15 mL fecal slurry and 50 mL of either baseline or raspberry ileal fluid were added to the fermentation vessel. The following conditions were maintained throughout the 24 h fermentation for ileal fluid samples; anaerobiosis, 37 °C, constant stirring, pH ~6.6. Aliquots of fermentates were collected before (0 h) and after (24 h) fecal fermentation, centrifuged at 13,500 rpm at 4 °C for 5 min and the supernatant filtered through 0.22 μm filters before being aliquoted and stored at −80 °C.

### 2.4 UHPLC-HR-MS analysis of (poly)phenols in ileal fluids and fermented samples

Extraction of (poly)phenolic compounds from ileal fluid and fermentates used a previously described method [23] and were analysed using a Dionex Ultimate 3000 RS UHPLC system comprising of a UHPLC pump, a PDA detector scanning from 200 to 600 nm and an autosampler operating at 4 °C (Thermo Scientific, San Jose, CA, USA). Separation of anthocyanins, ellagitannins and ellagic acids were derivatives was carried out using a 100 × 2.1 mm i.d. 1.8 μm Zorbax SB C18 column (Agilent Technologies, Berkshire, U.K.) maintained at 40 °C and eluted at a flow rate of 0.2 mL/min with a 50 min gradient of 3–70% 0.1% acidic methanol in 0.1% aqueous formic acid. After passing through the flow cell of the PDA detector the eluate was directed to an Exactive™ Orbitrap mass spectrometer fitted with a heated electrospray ionization probe (Thermo Scientific) operating in either negative ionization mode for the analysis of ellagitannins and gallic acids or in positive ionization mode for the anthocyanins. In negative ionization mode, analyses were based on scanning from 100 to 1000 *m/z*, with in-source collision-induced dissociation at 25.0 eV. The capillary temperature was 350 °C, the heater temperature was 150 °C, the sheath gas and the auxiliary gas flow rate were set at 25 and 5 units, respectively, and the sweep gas was 4 and the spray voltage was 3.00 kV. In positive ionization mode, analyses were based on scanning from 100 to 1000 *m/z*, with in-source collision-induced dissociation at 20.0 eV. The capillary temperature was 300 °C, the heater temperature was 150 °C, the sheath gas and the auxiliary gas flow rate were 40 and 10 units, respectively, and the sweep gas was 4 and the spray voltage was 3.00 kV. Data acquisition and processing were carried out using Xcalibur 3.0 software.

Identification was achieved by comparing the exact mass and the retention time with available standards, or by comparison with theoretical exact mass of the molecular ion with the measured accurate mass of the molecular ion, when standards were unavailable (see Table S1 in the Supplementary Information). Anthocyanins were quantified based on the theoretical exact mass of the molecular ion by reference to 0.05–100 ng/μL standard curves of cyanidin-3-*O*-glucoside and pelargonidin-3-*O*-glucoside. Ellagitannin and ellagic acid quantifications were carried out selecting the theoretical exact mass of the molecular ion by reference to 0.01–100 ng/μL standard curves of a mix of punicalagin and ellagic acid. A linear response was obtained for all the standard curves, as checked by linear regression analysis (R^2^ > 0.999). Limits of detection (ranging from 0.1 to 0.5 ng), limits of quantification (0.3–1.8 ng) and precision of the assay (as the coefficient of intra-assay variation, ranging from 1.5 to 4.7%) were considered acceptable, allowing the quantification of (poly)phenol compounds.

### 2.5 GC-MS analysis of phenolic acid catabolites in fermented samples

Extraction of phenolic compounds from ileal fluid and fermentates was performed using procedures described by Pereira-Caro et al. [8]. The phenolic catabolites from purified samples were analysed on a 6890 gas chromatograph equipped with a 7683S autosampler, a 5973 mass spectrometer (Agilent Technologies) and a ZB-5MS Zebron 30 m × 0.25 mm x 0.25 μm (i.d.) capillary column (Phenomenex, Macclesfield, Cheshire, U.K.) with helium as a carrier gas (1.2 mL/min). The GC-MS conditions were: injection volume (1 μL), split ratio (1:25), initial temperature 40 °C raised to 160 °C at 20 °C/min, to 200 °C at 1.5 °C/min, to 250 °C at 10 °C/min and a final temperature of 300 °C at 40 °C/min, which was held for 5 min. Injector, MS transfer line and ion source were maintained at 22 °C, 150 °C, and 230 °C, respectively. Mass spectra were scanned at *m/*z 50–470 and ionization energy of 70 eV. Phenolic compounds were identified according to retention time and mass spectra of authentic standards. Quantification was based on calibration curves of the ratio between the *m/z* of the target ion of the standard of interest and the target ion of the 2′,4′,5′-trimethoxycinnamic acid internal standard. For further information on the analytical method see Table S2.

### 2.6 Biological activity assays

#### 2.6.1 Tissue culture

Normal human epithelial colon cells, (CCD 841 CoN) [39] acquired from the American Type Culture Collection were incubated at 37 °C with 5% CO_2_ and grown as monolayers in roux flasks. Cells were sub-cultured every 3–4 days by the addition of trypsin (0.25% trypsin-EDTA) at 37 °C for 5 min. Cells were centrifuged at 1200 rpm for 3 min, the supernatant decanted, and cells resuspended in growth media. Cells were exposed to either ileal fluid fermentates or one of four phenolic acids, namely 3-(3′-hydroxyphenyl)propanoic acid, 3-(phenyl)propanoic acid, 4-hydroxybenzoic acid, and benzoic acid at 10, 50, and 100 μM. A 5 mM stock solution for each phenolic was prepared in 0.5% DMSO in PBS and subsequently diluted to test range in growth media to 0.01% DMSO.

*Exposure to treatments*. Cell viability was assessed via the MTT assay [40]. Due to the limited volume of ileal fluid fermentates available, a sample from a single participant was assessed at up to 40% v/v which establish a sub-cytotoxic dose of 20%. Cell viability was also established for the phenolic acids at 10, 50, and 100 μM.

For all experiments CCD 841 CoN cells were incubated with ileal fluids (20% v/v media) or phenolic acids at 10, 50, or 100 μM. An exposure time of 24 h was selected for all *ex vivo* studies, as it is generally considered to reflect the average colonic transit time.

#### 2.6.2 Genotoxicity assay

The COMET assay was conducted to determine the effect on DNA damage on normal epithelial colonic cells by 0 h and 24 h fermented ileal fluids at 20% (v/v in media) and by phenolic acids at 10, 50, or 100 μM, as outlined by De Santiago et al. [40]. Briefly, CCD 841 CoN cells were incubated with the various treatments for 24 h, prior to an oxidative challenge with 25 μM hydrogen peroxide or PBS. Results were acquired and analysed using Nikon eclipse 600 epifluorescent microscope and Komet 5.0 software (Kinetic Image ltd, Liverpool, UK). A total of 50 cells per gel were scored with a mean % tail DNA calculated from triplicate gels; each experiment was repeated three times, independently.

### 2.7 RNA isolation and cDNA synthesis

Following the method of McDougall et al. [41], after incubation for 24 h with one of the various treatments 841 CoN cells were collected, centrifuged, and the cell pellet homogenised using QIAshredder (Qiagen, UK). RNA was extracted with RNeasy Mini Plus Kit (Qiagen, UK) and its quality determined. The anchored-oligo(dT)_18_ and Transcriptor First Strand cDNA synthesis Kit (Roche) were used to obtain 20 μL of cDNA from 1 μg of RNA and its specificity based on the hypoxanthine phosphoribosyl transferase (HPRT) gene was determined via Lightcycler 480II (Roche). Following positive result, the cDNAs were stored at −20 °C.

### 2.8 Real-time PCR (qPCR)

An adapted version of the method of McDougall et al. [41] was used for primers design, qPCR performance and results analysis. In brief, OligoPerfect (Thermo Fisher, UK) and NCBI Primer-BLAST (USA) were used to design the primers for three genes belonging to the Nrf2-ARE pathway; Nuclear factor erythroid 2-related factor 2 (Nrf2), Heme oxygenase 1 (HO-1), and NAD(P)H dehydrogenase quinone 1 (NQO1). Genes and primers information are summarised in Table S3. The expression of each target cDNA sample was normalised using two housekeeping genes (*HPRT* and *β-actin*) and calculated as a ratio of the untreated control samples. All target cDNA samples were run as technical triplicates.

### 2.9 Statistical analysis

The mean of three independent experiments ± standard deviation (SDM) was calculated for each type of dataset and used for the statistical analysis. The Shapiro-Wilk test was used to determine normality. Paired-samples *t*-test, for COMET ileal fluid results, and One-way ANOVA and Dunnett's Multiple comparison post-hoc test, for results from phenolic compounds and gene expression studies, were used to assess significant differences between means (significance levels p < 0.05). Analyses were performed using IBM SPSS Statistics for Windows (version 25.0, 2017. Armonk, NY: IBM Corp).

Analyses of (poly)phenol content were performed in triplicate. Data are reported as mean values ± standard error (SEM). One-way ANOVA was performed to evaluate significant differences between metabolites presented before and after fermentation (significance levels p < 0.05). The statistical analysis was performed using R software (version 3.5.0).

Multivariate statistical analysis (Multilevel PLS-DA, Partial Least Square Discriminant Analysis), where the between subject variation is separated from the within subject variation, was used for selection of the most discriminative metabolites between groups. Models were optimized based on a leave-one out cross validation. Variable Importance in Projection (VIP) scores were obtained and only metabolites that exceeded an initial threshold, set to 0.9, were selected. All these analyses were carried out using R software (version 3.5.0) and the Solo software (version 8.7, 2019. Eigenvector Research Inc., Manson, WA, USA).

## 3 Results

### 3.1 Characterization of the ileal fluids before and after fecal fermentation

The (poly)phenolic composition of the ingested 300 g of raspberries has been described previously (see Table S4), as have the levels recovered in ileal fluid collected 0–8 h after raspberry consumption [41]. In the current study these ileal fluid samples from 11 subjects were subjected to *ex vivo* fecal fermentation, with samples taken pre- (0 h) and post-fermentation (24 h) being analysed by UHPLC-HRMS and GC-MS. The changes in the amounts of key anthocyanins, ellagic acid derivatives and ellagitannins following fermentation are presented in Table 1. Substantial inter-individual variation was evident in the (poly)phenolic composition of the raspberry ileal fluid before fermentation (0 h). The amounts of the major ellagitannins varied considerably with sanguiin H-6 levels of 0.6–35.1 μmol and sanguiin H-10 of between 1.0 and 10.0 μmol. The most abundant anthocyanin, cyanidin-3-*O*-sophoroside ranged from 0.6 to 10.0 μmol and the least abundant anthocyanins, cyanidin-3-*O*-glucoside (n.d. - 0.3 μmol) and pelargonidin-3-*O*-sophoroside (n.d. - 0.1 μmol) also showed inter-individual variation. However after 24 h fecal fermentation, the quantities in the raspberry ileal fluids decreased, with cyanidin-3-*O*-sophoroside at 0.2–4.0 μmol, sanguiin H-6 at 0.1–3.3 μmol, sanguiin H10 at 0.1–0.9 μmol, cyanidin-3-*O*-glucoside (n.d. - 0.1 μmol) and pelargonidin-3-*O*-sophoroside (n.d. - 0.04 μmol). Ellagic acid was also reduced from 1.2 - 20.0 μmol to 1.1–11.1 μmol, but the decreases (8.3–44.5%) were much less severe than those of the anthocyanins and ellagitannins. In context of the original quantity of raspberry (poly)phenols consumed, only 7% of the ingested dose of cyanidin-3-*O*-sophoroside was recovered at the end of the simulated colonic fermentation, less than 2% of ingested sanguiin H-6 and H-10 but 109% of ingested ellagic acid (Table 2). The increase in ellagic acid is due to its release from ellagitannins and ellagic acid glycosides in the ileum and continued degradation of these components by the fecal microflora, which maintained colonic ellagic acid availability.

**Table 1:** Quantities of (poly)phenols in ileal fluid from subjects who consumed 300 g of raspberries before (0 h) and after (24 h) fecal fermentation. Data expressed as μmol ± SDM (n = 3)^a^.

Phenolic compounds	S1-0 h	S1-24 h	S2-0 h	S2-24 h	S4-0 h	S4-24 h	S5-0 h	S5-24 h	S6-h	S6-24 h
Cyanidin-3-*O*-sophoroside	6.4 ± 0.5^a^	1.8 ± 0.2^b^	3.3 **±** 0.3^a^	1.1 **±** 0.1^b^	5.2 ± 0.6^a^	1.9 ± 0.2^b^	0.6 ± 0.1^a^	0.2 ± 0.1^b^	5.0 ± 1.2^a^	1.6 ± 0.4^b^
Cyanidin-3-*O*-(2"-*O*-glucosyl)rutinoside	1.3 ± 0.4^a^	0.5 ± 0.1^b^	1.2 **±** 0.1^a^	0.4 **±** 0.0^b^	1.3 ± 0.2^a^	0.4 ± 0.1^b^	0.2 ± 0.1^a^	0.1 ± 0.0^b^	1.2 ± 0.3^a^	0.4 ± 0.1^b^
Pelargonidin-3-*O*-sophoroside	0.1 ± 0.0^a^	0.03 ± 0.02^b^	0.1 **±** 0.0^a^	0.02 **±** 0.00^b^	0.1 ± 0.0^a^	0.02 ± 0.00^b^	n.d.	n.d.	0.1 ± 0.0^a^	0.02 ± 0.01^b^
Cyanidin-3-*O*-glucoside	0.06 ± 0.04^a^	0.02 ± 0.01^b^	0.3 **±** 0.0^a^	0.1 **±** 0.0^b^	0.1 ± 0.0^a^	0.03 ± 0.01^b^	n.d.	n.d.	0.1 ± 0.0^a^	0.02 ±0.00^b^
Cyanidin-3-*O*-rutinoside	0.2 ± 0.0^a^	0.1 ± 0.0^b^	0.1 **±** 0.0^a^	0.03 **±** 0.00^b^	n.d.	n.d.	0.1 ± 0.1^a^	0.1 ± 0.0^a^	0.2 ± 0.0^a^	0.1 ± 0.0^b^
Total anthocyanins	8.1 ± 0.9^a^	2.4 ± 0.3^b^	5.0 ± 0.4^a^	1.6 ± 0.1^b^	6.7 ± 0.8^a^	2.3 ± 0.3^b^	0.9 ± 0.3^a^	0.4 ± 0.1^b^	6.6 ± 1.5^a^	2.1 ± 0.5^a^
Ellagic acid pentoside	0.2 ± 0.1^a^	0.04 ± 0.02^b^	0.5 ± 0.1^a^	0.1 ± 0.0^b^	0.4 ± 0.2^a^	0.1 ± 0.1^b^	0.1 ± 0.1^a^	0.1 ± 0.0^b^	0.4 ± 0.2^a^	0.1 ± 0.1^a^
Ellagic acid	3.8 ± 0.5^a^	3.6 ± 0.4^a^	6.2 ± 2.3^a^	4.8 ± 2.1^a^	9.5 ± 4.8^a^	8.0 ± 4.0^a^	1.2 ± 0.1^a^	1.1 ± 0.1^a^	9.5 ± 2.3^a^	6.2 ± 1.6^b^
Total ellagic acid	4.0 ± 0.6^a^	3.64 ± 0.42^a^	6.7 ± 2.4^a^	4.9 ± 2.1^a^	9.9 ± 5.0^a^	8.1 ± 4.1^a^	1.3± 0.2^a^	1.2 ± 0.1^a^	9.9 ± 2.5^a^	6.3 ± 1.7^b^
Sanguiin H-10	9.4 ± 0.5^a^	0.9 ± 0.0^b^	4.7 ± 0.6^a^	0.5 ± 0.1^b^	5.2 ± 0.2^a^	0.3 ± 0.0^b^	1.0 ± 0.1^a^	0.1 ± 0.0^b^	6.4 ± 1.4	0.5 ± 0.1^b^
Sanguiin H-6	4.7 ± 0.7^a^	0.5 ± 0.1^b^	7.5 ± 3.2^a^	0.7 ± 0.3^b^	23.0 ± 2.5^a^	2.0 ± 0.2^b^	0.6 ± 0.1^a^	0.1 ± 0.0^b^	15.0 ± 2.3^a^	1.3 ± 0.2^b^
Lambertianin C	n.d.	n.d.	0.5 ± 0.6^a^	0.1 ± 0.1^b^	3.3 ± 1.6^a^	0.2 ± 0.1^b^	n.d.	n.d.	n.d.	n.d.
Total ellagitannins	14.1 ± 1.1^a^	1.4±0.1^b^	12.7 **±** 4.4^a^	1.3 ± 0.5^b^	31.5 ± 4.3^a^	2.5 ± 0.3^b^	1.6 ± 0.2^a^	0.2 ± 0.0^b^	21.4 ± 3.7^a^	1.8 ± 0.3^b^
*Total (poly)phenols*	*26.2* ± 2.6^a^	*7.4*±0.8^b^	*24.4* **±** 7.2^a^	*7.8* **±** 2.7^b^	*48.1* ± 10.1^a^	*12.9* ± 4.7^b^	*3.8* ± 0.7^a^	*1.8* ± 0.2^b^	*37.9* ± 7.7^a^	*10.2* ± 2.5^b^

(Poly)phenolic compounds	S9-0 h	S9-24 h	S10-0 h	S10-24 h	S11-0 h	S11-24 h	S12-0 h	S12-24 h
Cyanidin-3-*O*-sophoroside	10.0 ± 0.3^a^	4.0 ± 0.1^b^	5.1 ± 0.5^a^	1.8 ± 0.1^b^	2.9 ± 0.6^a^	1.0 ± 0.2^b^	1.5 ± 0.6^a^	0.5 ± 0.2^b^
Cyanidin-3-*O*-(2"-*O*-glucosyl)rutinoside	2.4 ± 0.0^a^	0.8 ± 0.0^b^	1.2 ± 0.0^a^	0.4 ± 0.0^b^	0.7 ± 0.3^a^	0.2 ± 0.1^b^	0.4 ± 0.1^a^	0.2 ± 0.1^b^
Pelargonidin-3-*O*-sophoroside	0.1 ± 0.0^a^	0.04 ± 0.00^b^	0.1 ± 0.0^a^	0.02 ± 0.00^b^	0.1 ± 0.0^a^	0.01 ± 0.00^b^	n.d.	n.d.
Cyanidin-3-*O*-glucoside	0.1 ± 0.0^a^	0.03 ± 0.00^b^	0.1 ± 0.0^a^	0.02 ± 0.00^b^	0.1 ± 0.1^a^	0.1 ± 0.1^b^	0.1 ± 0.0^a^	0.01 ± 0.01^b^
Cyanidin-3-*O*-rutinoside	0.2 ± 0.03^a^	0.1 ± 0.0^b^	0.7 ± 0.1^a^	0.3 ± 0.0^b^	0.01 ± 0.01^a^	0.01 ± 0.01^b^	0.2 ± 0.1^a^	0.1 ± 0.0^b^
Total anthocyanins	12.8 ± 0.3^a^	5.0 ± 0.1^b^	7.2 ± 0.6^a^	2.5 ± 0.1^b^	3.8 ± 1.0^a^	1.3 ± 0.4^b^	2.2 ± 0.8^a^	0.8 ± 0.3^b^
Ellagic acid pentoside	0.6 ± 0.1^a^	0.1 ± 0.0^b^	0.4 ± 0.1^a^	0.1 ± 0.0^b^	0.1 ± 0.0^a^	0.02 ± 0.00^b^	0.4 ± 0.1^a^	0.1 ± 0.0^b^
Ellagic acid	20.0 ± 2.1^a^	11.1 ± 1.2^b^	7.1 ± 1.0^a^	5.3 ± 0.8^a^	5.8 ± 0.5^a^	4.2 ± 0.3^b^	2.9± 1.4^a^	2.6 ± 0.8^a^
Total ellagic acid	20.6 ± 2.2^a^	11.2 ± 1.2^b^	7.5 ± 1.1^a^	5.4 ± 0.8^a^	5.9 ± 0.5^a^	4.2 ± 0.3^b^	3.3 ± 1.5^a^	2.7 ± 0.8^a^
Sanguiin H-10	10.0 ± 2.7^a^	0.8 ± 0.2^b^	6.0 ± 0.2^a^	0.5 ± 0.0^b^	4.7 ± 0.6^a^	0.5 ± 0.1^b^	2.1 ± 0.8^a^	0.1 ± 0.1^b^
Sanguiin H-6	35.1 ± 3.3^a^	3.3 ± 0.3^b^	15.3 ± 5.0^a^	1.5 ± 0.5^b^	9.4 ± 1.2^a^	0.9 ± 0.1^b^	1.2 ± 0.3^a^	0.1 ± 0.0^b^
Lambertianin C	5.4 ± 0.7^a^	0.4 ± 0.1^b^	1.8 ± 0.5^a^	0.1 ± 0.0^b^	1.2 ± 0.7^a^	0.1 ± 0.1^b^	n.d.	n.d.
Total ellagitannins	50.5 ± 6.7^a^	4.5 ± 0.6^b^	23.1 ± 5.7^a^	2.1 ± 0.5^b^	15.3 ± 2.5^a^	1.5 ± 0.3^b^	3.3 ± 1.1^a^	0.2 ± 0.1^b^
*Total (poly)phenols*	*83.9 ± 9.4*^*a*^	*20.7 ± 1.9*^*b*^	*37.8 ± 7.4*^*a*^	*10.0 ± 1.4*^*b*^	*25 ± 4*^*a*^	*7 ± 1*^*b*^	*8.8 ± 3.4*^*a*^	*3.7 ± 1.2*^*b*^

a Different letters denote significant different within the same subject (p < 0.05). Can this be made standard error rather than SD. Insufficient sample available for the analysis for participants S03 and S08.

**Table 2:** Quantities of anthocyanins, ellagic acid derivatives and ellagitannins recovered in A) ileal fluids collected 0–8 h after raspberry consumption and in B) raspberry ileal fluid after 24 h fecal fermentation. Data expressed as μmol ± SEM (for A n = 11, for B n = 3).

Compounds	A-Recovery after small intestine		B-Recovery after fecal fermentation	
	μmol ±SEM	% Intake	μmol ±SEM	% Intake
Cyanidin-3-*O*-sophoroside	4.7 ± 0.8	21.4	1.5 ± 0.1	7.0
Cyanidin-3-*O*-(2"-*O*-glucosyl)rutinoside	1.2 ± 0.3	28.6	0.37 ± 0.03	8.9
Pelargonidin-3-*O*-sophoroside	0.06 ± 0.02	24.0	0.02 ± 0.001	6.7
Cyanidin-3-*O*-glucoside	0.11 ± 0.04	1.2	0.03 ± 0.003	0.3
Cyanidin-3-*O*-rutinoside	0.23 ± 0.06	10.5	0.07 ± 0.01	3.4
**Total anthocyanins**	**6.3 ± 1.2**	**16.5**	**2.0 ± 0.2**	**5.3**
Ellagic acid-*O*-pentoside	0.4 ± 0.1	33.3	0.06 ± 0.003	4.9
Ellagic acid	8.4 ± 1.5	175	5.2 ± 0.3	109
**Total ellagic acid**	**8.8 ± 1.6**	**147**	**5.3 ± 0.3**	**88**
Sanguiin H-10	6.0 ± 1.3	14.0	0.47 ± 0.03	1.1
Sanguiin H-6	18.7 ± 4.9	30.7	1.16 ± 0.12	1.9
Lambertianin C	1.6 ± 0.9	8.0	0.11 ± 0.12	0.6
**Total ellagitannins**	**26 ± 7**	**21.2**	**1.7 ± 0.2**	**1.4**

**Fig. 1:**
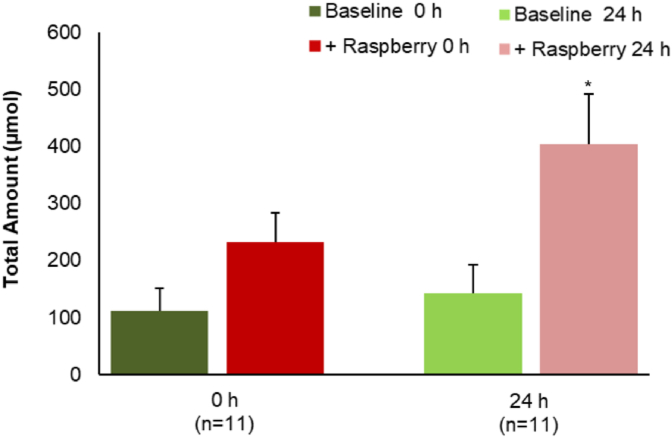
Total amount of phenolic acids and aromatic catabolites present in ileal fluid before (baseline) and after (+raspberry) raspberry consumption analysed before (0 h) and after (24 h) fecal fermentation. Data expressed as μmol ± SEM (n = 11). Significance compared Baseline vs + Raspberry using One-way ANOVA and Dunnett T test, **p < 0.05.

**Fig. 2:**
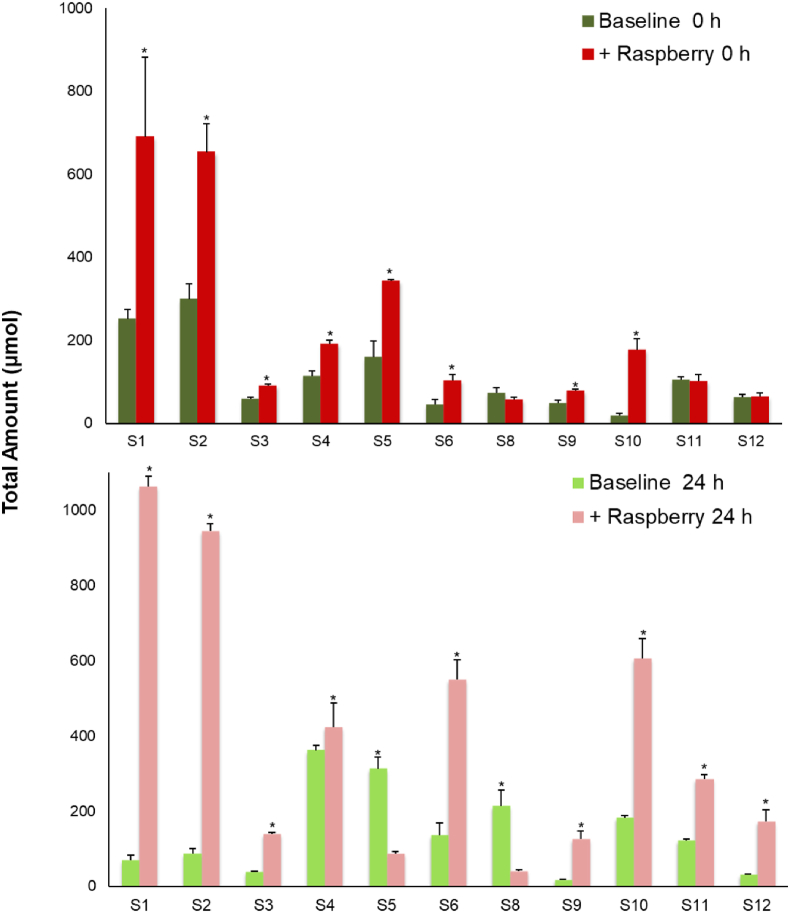
The impact of fecal fermentation on the total amount of phenolic acid and aromatic catabolites in ileal fluid collected before (Baseline) and after the consumption of 300 g of raspberry puree (+Raspberry) by 12 subjects. Baseline and +Raspberry samples before fecal fermentation (0 h). Baseline and +Raspberry samples after 24 h fecal fermentation (24 h). Significance compared for each subject between pre- and post-berry intake using One-way ANOVA and Dunnett T test, *p < 0.05.

In contrast to the substantial decrease in the amounts of most (poly)phenols (Table 2), total phenolic acids and aromatic catabolites increased significantly (~30%, p < 0.005) in the raspberry ileal fluids after 24 h fermentation (Fig. 1). Details of the volunteer-volunteer variations are presented in Fig. 2. A total of 17 phenolic acid catabolites were detected and quantified in the raspberry ileal fluid samples before and after fermentation (Table S5) including benzoic acid (4.7–132 μmol), 3-(3′-hydroxyphenyl)propanoic acid (n.d. - 8 μmol), 3-(phenyl)propanoic acid (n.d. - 19 μmol), and, 4-hydroxybenzoic (n.d. - 26 μmol).

Based on the data in Table S5 and the voided volumes of 0–8 h ileal fluid, it is noteworthy that the concentration, as opposed to the quantity of (poly)phenols in the eleven 0-h pre-fermented raspberry ileal fluid samples ranged from 55 to 1668 μM (mean 581 ± 171 μM) while the 24-h post-fermented samples contained 162–1962 μM (mean 807 ± 187 μM) (Table 3). Information on the concentration of the 17 individual phenolic acids is presented in Table S6.

**Table 3:** Concentration of anthocyanins, ellagic acid derivatives, ellagitannins and low molecular weight phenolics recovered in ileal fluids collected 0–8 h after raspberry consumption and in raspberry ileal fluid after a 24 h fecal fermentation. Data expressed as μM ± SEM (n = 11)^a^.

Compounds	Concentration in ileal fluid		Concentration in ileal fluid after fecal fermentation	
	μM ± SEM	Range (μM)	μM ± SEM	Range (μM)
**Total anthocyanins**	7.1 ± 0.9	3.0–10.2	2.4 ± 0.3*	1.0–4.0
**Total ellagic acids**	9.7 ± 1.7	2.8–16.4	6.9 ± 1.0	2.6–10.7
**Total ellagitannins**	23.6 ± 4.9	5.3–41.9	2.1 ± 0.4*	0.3–3.7
3-(3′-Hydroxyphenyl)propanoic acid	9 ± 1	n.d. - 13.0	25 ± 9**	n.d. - 84
3-(Phenyl)propanoic acid	6 ± 3	0.7–33	28 ± 14*	n.d. - 144
4-Hydroxybenzoic acid	26 ± 9	n.d. - 87	21 ± 5	4–51
Benzoic acid	76 ± 20	14–246	149 ± 28*	30–357
**Total phenolics**^b^	581 ± 171	55–1668	807 ± 187**	162–1962

a Data on all of the individual phenolics detected in the samples and listed in Table S5.

b The mean value post-fermentation is statistically different compared with the mean value pre-fermentation. One-way ANOVA and Dunnett T test, (*) p < 0.05; (**) p < 0.005.

**Fig. 3:**
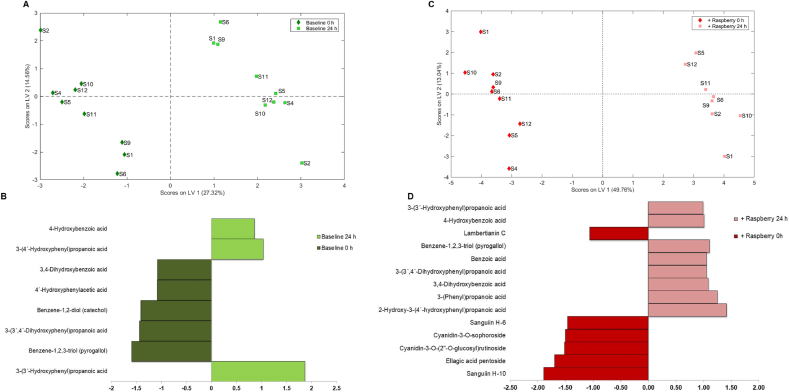
Multi-Level Partial Least Squares-Discriminant Analysis (ML-PLSDA) plots of phenolic composition of baseline ileal samples pre-raspberry consumption (A and B) and post-raspberry consumption (C and D) before (red) and after (green) 24 h of fecal fermentation. Score plots are Figures A and C and loadings weights plot for component 1 are Figures B and D. Cut off VIP value set at 0.9. (For interpretation of the references to color in this figure legend, the reader is referred to the Web version of this article.)

Although the fecal microbiota donor was constant across each of the gut models, high inter-person variation was evident in the concentrations of the catabolites detected. To overcome this variation and to obtain a clearer picture of the impact of fecal fermentation on the phenolic composition of ileal fluid(s) a multivariate approach (multilevel partial least squares-discriminant analysis, ML-PLS-DA) was carried out (Fig. 3). As shown in Fig. 3A there was a clear differentiation between the pre- and post-fermentation ileal fluid samples, with an average classification error of 0.00, which accounted for 27.3% of total variance (latent variable 1, LV1) (Fig. 3A). Eight phenolic acid catabolites were indicated as the key discriminators of the fermentation of the baseline ileal fluid samples (Fig. 2B) with significant increases in 3-(4′-hydroxyphenyl)propanoic acid, 4-hydroxybenzoic acid and 3-(3′-hydroxyphenyl)propanoic acid. Fourteen key compounds were identified, with LV1 accounting for 48.7% of total variance, which discriminated between the before and after fermentation states (Fig. 3C and D). Raspberry (poly)phenolics including sanguiin H-6, sanguiin H-10 and cyanidin-3-*O*-sophoroside decreased significantly during fermentation of raspberry ileal fluid, while low molecular weight phenolics including 3-(3′,4′-dihydroxyphenyl)propanoic acid, 3-(3′-hydroxyphenyl)propanoic acid, 4′-hydroxyphenylactic acid, 3-phenylacetic acid, 3,4-dihydroxybenzoic acid, 4-hydroxybenzoic acid, benzoic acid, and benzene-1,2,3-triol significantly increased. The microbial catabolites benzoic acid, 4-hydroxybenoic acid, and 3-(3′-hydroxyphenyl)propanoic acid were identified as discriminants of fermented raspberry ileal fluids (Fig. 3D) and, along with 3-(phenyl)propanoic acid, were used for subsequent bioactivity studies.

### 3.2 Bioactivity of ileal fluid fermentates and microbial metabolites

#### 3.2.1 Ileal fluid fermentates

**Fig. 4:**
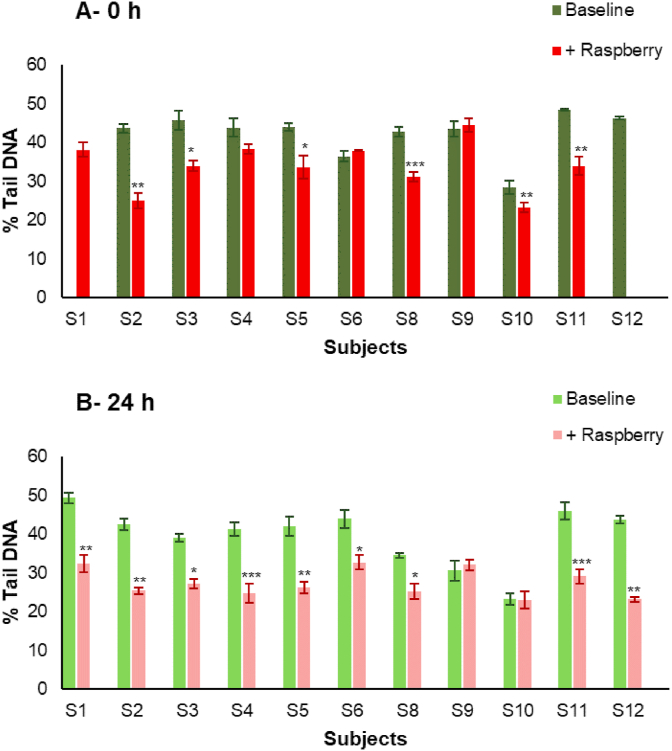
Effects on DNA damage of ileal fluid fermentates (IFFs). A) Baseline and +Raspberry ileal fluids after 0 h fermentation and B) Baseline and +Raspberry ileal fluids after 24 h fermentation (at 20% ileal fluids (v/v) in growth media). After 24 h pre-incubation at 37 °C, CoN CCD 841 cells were challenged with 25 μmol/L H_2_O_2_. Data expressed as mean of three independent experiments ± SDM. Significance is compared to Baseline per each subject using Paired-sample *t*-test, *p < 0.05, **p < 0.01, ***p < 0.001.

For the majority of participants, the fermented raspberry ileal fluids exerted a significantly greater reduction of DNA damage than the pre-fermented condition (Fig. 4A). At 0 h, 6 out of the 11 raspberry ileal fluids (Fig. 4A; participants S2, S3, S5, S8, S10 and S11), significantly decreased % Tail DNA compared to their baseline counterparts. After a 24 h fermentation, 9 out of 11 raspberry ileal fluids (Fig. 3B; participants S1, S2, S3, S4, S5, S6, S8, S11 and S12), exerted significantly greater reduction of DNA damage than the baseline samples. Moreover, the ileal fluid fermentates themselves, without an oxidative challenge, exhibited little inherent DNA damaging activity (average 5.9 ± 2.7% Tail DNA), while the average % Tail DNA for experimental controls (media only) after challenge with H_2_O_2_ or PBS, was 50.8 ± 3.5 and 4.1 ± 0.2 respectively.

The Nuclear factor erythroid 2-related factor 2 (Nrf2) regulate genes encoding NAD(P)H dehydrogenase, quinone-1 (NQO1) and heme oxygenase-1 (HO-1), reduce reactive oxygen species and play a key role in cytoprotection. Raspberry ileal fluid samples generally increased target gene expression (, NQO1 and HO-1) while the baseline samples down-regulated these genes (Fig. 4). Nrf2 expression was significantly down-regulated (≥1.04-fold change, p < 0.001 vs control) by 8 out of 11 baseline ileal fluids (participants S1, S2, S3, S4, S5, S6, S8 and S10). Expression of NQO1 was significantly down-regulated, at least 50% (p < 0.001), by all baseline ileal samples with the greatest decrease following incubation with S5 (2.27-fold, p < 0.001). Significant reductions (p < 0.01) in HO-1 expression ranging from 1.07-fold to 1.64-fold were observed after treatment with 7 out of 11 baseline samples (participants S1, S2, S8, S9, S10, S11 and S12).

**Fig. 5:**
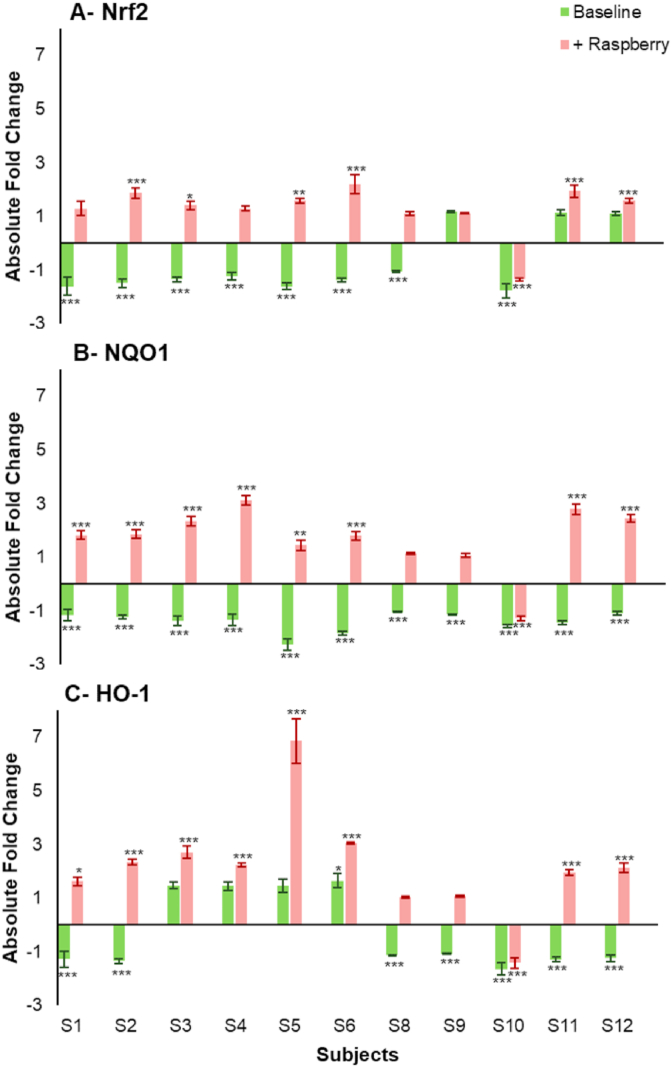
Changes in (A) Nrf2, (B) NQO1, (C) HO-1 gene expression after incubation for 24 h with Baseline and +Raspberry ileal fluid fermented for 24 h (20% v/v). Gene expression in CCD841 cells is presented as mean of 3 independent experiments ±SDM and expressed as absolute fold change. Significance is compared to normalised untreated control using One-way ANOVA and Dunnett's Multiple Comparisons test. *p < 0.05, **p < 0.01, ***p < 0.001.

On the other hand, the raspberry ileal fluids generally caused increases in expression of target genes, apart from samples for participant S10. For Nrf2, 10 of 11 raspberry samples caused increases in expression with 6 (participants S2, S3, S5, S6, S11 and S12) being significant (Fig. 5). For NQO1, 8 raspberry samples showed significant increases in expression (participants S1, S2, S3, S4, S5, S6, S11 and S12) while for HO-1, 8 samples showed significant increases in expression with sample S5 causing a 6.87-fold increase (p < 0.001).

### 3.3 Phenolic catabolites

**Fig. 6:**
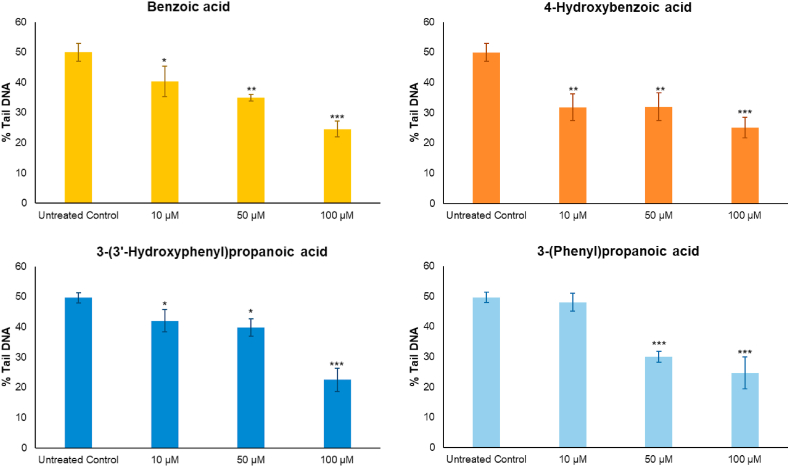
Effects on DNA damage of four phenolic compounds. Components were tested after 24 h pre-incubation at 37 °C, CoN CCD 841 cells were challenged with 25 μM H_2_O_2_. Data expressed as mean of 3 independent experiments ± SDM. Benzoic acid, 4-hydroxybenzoic acid, 3-(3′-hydroxyphenyl)propanoic acid, 3-(phenyl)propanoic acid, at 10, 50, or 100 μM. Significance is compared to baseline (i.e. Untreated Control) using One-way ANOVA and Dunnett's Multiple Comparisons test. *p < 0.05, **p < 0.01, ***p < 0.001.

**Fig. 7:**
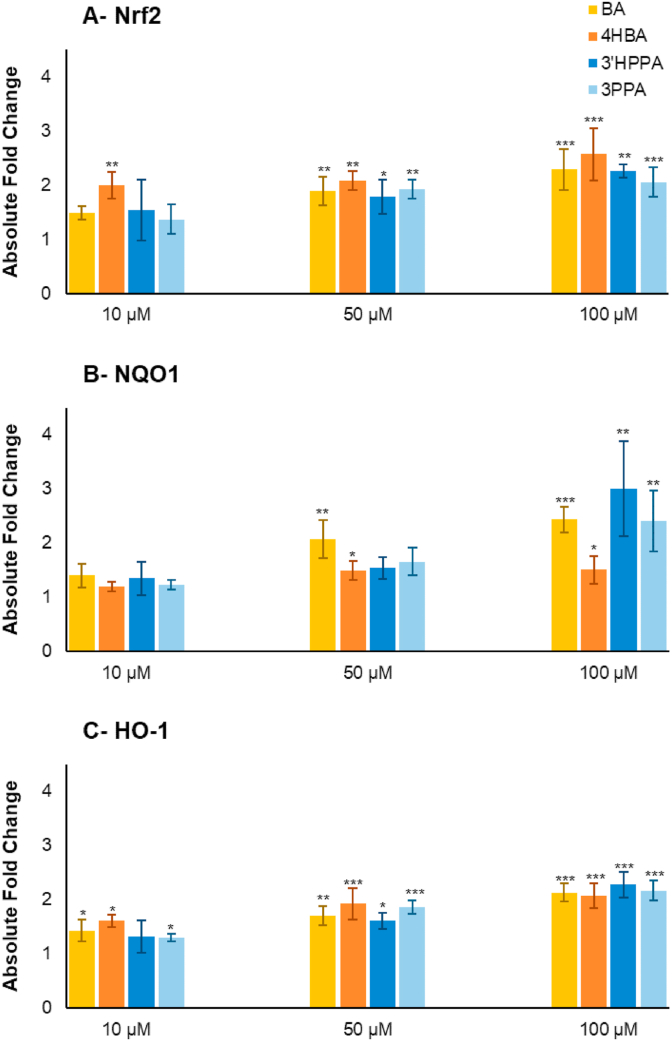
Change in (A) Nrf2, (B) NQO1, (C) HO-1 gene expression after pre-incubation with four phenolic compounds. Benzoic acid (BA), 4-hydroxybenzoic acid (4HBA), 3-(3′-hydroxyphenyl)propanoic acid (3′HPPA), and 3-(phenyl)propanoic acid (3PPA). Absolute fold change in expression is presented as mean of 3 independent experiments ±SDM on CCD 841 CoN cells. Significance is compared to normalised untreated control using One-way ANOVA and Dunnett's Multiple Comparisons test. *p < 0.05, **p < 0.01, ***p < 0.001.

The microbial catabolites benzoic acid, 4-hydroxybenzoic acid, and 3-(3′-hydroxyphenyl)propanoic acid were identified as discriminants of raspberry fermented ileal fluids (Fig. 3D). These were assessed at a sub-cytotoxic dose range (data not shown) for DNA damage activity. 3-(Phenyl)propanoic acid was also assessed as a non-hydroxylated comparator to 3-(3′-hydroxyphenyl)propanoic acid. Pre-treatment with all these phenolic acids at concentrations comparable with the those detected in some of the pre- and post-fermented ileal fluid (Table S5) decreased DNA damage induced by an oxidative challenge in a dose-dependent manner. At 100 μM, they reduced DNA damage by ~50% (p < 0.001) compared to the untreated control (Fig. 6) whereas at the lowest concentration (10 μM) all but 3-(phenyl)propanoic acid significantly (p < 0.05) decreased DNA damage by >20%. Concomitant with these effects, the phenolic acids also induced upregulation of expression of Nrf2, NQO1 and HO-1 genes in a dose-dependent manner with up to a 3-fold increase in expression (Fig. 7).

## 4 Discussion

The study of (poly)phenol rich foods on gut health has been hampered by the frequent testing of physiologically irrelevant compounds [[26], [27], [28], [29], [30], [31]]. More recently, the importance of the colonic microbiota in the biotransformation and consequently the bioactivity of (poly)phenols has been appreciated [[10], [11], [12], [13]] through evidence obtained predominantly through *ex vivo* fermentation models, using human fecal microbiomes to catabolise either whole foods, *in vitro* digested (poly)phenolic extracts [37,[42], [43]] or individual phenolic compounds [38,44]. In the current study use was made of ileal fluid, which provides a source of dietary compounds not absorbed by the small intestine that would ultimately become available for interaction with the gut microbiota and the colonic epithelium. The ileal fluid samples were subjected to *ex-vivo* fecal fermentation and analysed for changes in phytochemical composition, activity in protection against DNA damage and activation of the Nrf2-ARE pathway.

The recovery of ingested anthocyanins in ileal fluids after raspberry intake (e.g. anthocyanins 6.3 ± 1.2 μmol, ~16.5% of intake), was consistent with the recovery rates (11.7–40%) reported for other ileostomy bioavailability studies on berries [20,22,24]. A 24 h fecal fermentation of these samples, demonstrated significant catabolism of the raspberry (poly)phenols with a total mean recovery of only 5.4% of anthocyanins and 1.4% of ellagitannins compared with the amounts in the 300 g of ingested raspberries (Table 2, Fig. 2). This *ex vivo* study demonstrated the residual raspberry anthocyanins in ileal fluids were heavily catabolized by fecal microbiota (1.8–20.7 μmol, Table 1) and thereby contributed to the significant increase in the total concentration of phenolic and aromatic compounds from 581 ± 171 μM to 807 ± 187 μM (Table 3).

Indeed**,** anthocyanins that resist upper GI tract metabolism are deglycosylated and the released anthocyanidins subjected to fission of the C-ring, with the released A- and B-ring fragments converted to a broad range of microbial-derived phenolic acid and aromatic compounds [10,13]. For example, cyanidin is degraded *in vitro* by the colonic microbiota via B-ring fission to 3,4-dihydroxybenzoic acid and subsequently to 4-hydroxybenzoic acid, or alternatively transformed, to a lesser extent, to 3-(3′,4′-dihydroxyphenyl)propanoic acid and then converted to phenylpropanoic and phenylacetic acid derivatives [45]. Therefore, accumulation of these microbially-derived compounds (Tables 3 and S5) is likely a consequence of the colonic degradation of the raspberry anthocyanidins. In contrast, urolithins are microbially-derived from ellagitannins and ellagic acids [20,45], and these were absent in the raspberry ileal fluids. This is probably a consequence of the single fecal sample donor being a metabotype 0 lacking bacterial strains, such as *Gordonibacter* species, *G. urolithnfaciens* and *G. pamelaeae*, that are required for conversion of ellagitannins and ellagic acids to urolithins [46]. Comparing the *ex vivo* digestion model to commonly used *in vitro* digestions, key differences in either (poly)phenol composition and/or estimated concentration are found [47]. *In vitro* digestion procedures cannot fully mimic uptake through the action of the small intestine brush-border enzymes phlorizin-hydrolase and cytosolic β-glucosidases [13] nor any hepato-portal efflux, and thereby overestimates recovery. For example, the total parent anthocyanin recovery after *in vitro* digestion of a raspberry extract has been reported between 40 and 60% [47] rather than 16.5% observed in ileal fluid (Table 2) in the current study.

In the present study the *ex-viv*o approach offers an opportunity to evaluate (poly)phenol metabolites and catabolites in a physiologically relevant context, as all too often biological effects are assessed at supra-physiological concentrations (e.g. 20–40 μM of cyanidin-3-*O*-glucoside [48] or 120 μM of cyanidin-3-*O*-rutinoside [28], which contrasts to their maximum respective concentrations of 0.3 μM and 0.1 μM noted in the raspberry fermented ileal fluids (data converted from Table 1).

(Poly)phenols exert chemopreventive effects, in part by inhibiting DNA damage via modulation of antioxidant response pathways [32], which is important given that an excess of oxidants are able to alter the normal structure and function of DNA, lipids, and proteins, leading to mutations or oxidative damage. Following the consumption of raspberries, ileal fluids were characterised by the presence of high quantities of microbially-derived phenolic metabolites (162–1962 μM, Table 3) rather than the parent (poly)phenolic compounds (~12 μM; Table 3). Nine out the eleven raspberry ileal fluid fermenates exerted significantly greater reduction in DNA damage (~29%) than then their baseline counterparts (Fig. 4).

The microbial metabolites benzoic acid, 4-hydroxybenzoic acid and 3-(3′-hydroxyphenyl)propanoic acid were noted as being representative of the fermentation of the raspberry ileal fluids and were shown to have potency against DNA damage at concentrations observed within the fermentates (Table S5). Previously, 100 and 200 μM of benzoic acid was reported to be neuro-protective against oxidative challenges [49] while 50–1000 μM 5-*O*-caffeoylquinic acid (aka chlorogenic acid) and its microbial metabolites, 3′,4′-dihydroxycinnamic acid (aka caffeic acid), 3-(phenyl)propanoic acid and benzoic acid exerted anti-proliferative effects in Caco2 cells (50–1000 μM) and mixtures of these compounds increased potency [50].

Despite the different spectrum of phenolic catabolites in the pre-and post-fermented raspberry ileal fluids, no simple correlations with either total or individual phenolics could be derived to explain the greater DNA damage reduction of the fermented samples (Fig. 4B). The ileal samples reflect consumption of a whole food and consequently contain a complex mixture of (poly)phenolic compounds, as well other potential bioactive components. It is, therefore, possible that additive and synergistic interactions are responsible for the different patterns observed.

The cytoprotective effects noted are likely mediated through the Nrf2-ARE pathway and activation of downstream regulated target genes such as HO-1 or NQO1 either by affecting ROS or promotion of Nrf2 nucleus translocation by inhibition of ubiquitination Kelch-like ECH-associating protein 1 [32]. The majority (9 out of 11) of raspberry ileal fluids significantly increased (>2-fold) the expression of Nrf2, HO-1 or NQO1 in CCD 841 CoN cells, as did the selected microbial catabolites (10–100 μM). Modulation of gene expression in the Nrf2-ARE pathway in colonocytes has been reported for cyanidin-3-*O*-glucoside, albeit at high, 20–40 μM, concentrations which also significantly increased HO-1 and NQO1 in Caco2 cells [48]. Raspberry triterpenoids at a much lower 100 nM dose upregulated NQO1 and down regulated Nrf2 and HO-1 while reducing DNA damage in CCD 841 cells [41]. It is also noteworthy that anthocyanin-rich bilberry extract (*Vaccinium myrtillus* L.*)* consumption in humans upregulated NQO1 but decreased Nrf2 and HO-1 gene expression in peripheral blood mononuclear cells concomitant with DNA damage reduction. However, these benefits were not apparent in subjects lacking a colon (ileostomates), highlighting the importance of colonic catabolism in causing systemic changes [51].

## 5 Conclusions

Following consumption of raspberries, (poly)phenols that survive digestion in the small intestine enter the colon where they are subject to microbial catabolism. We have demonstrated that physiologically relevant raspberry-enriched ileal fluid fermentates reduce DNA damage in normal colonocytes mediated in part by the up-regulation of the cytoprotective Nrf2-ARE pathway driven by (poly)phenolic catabolites. Therefore, modifications of raspberry (poly)phenols in the GI tract result in the production of low molecular weight catabolites that may exert protective effects against colonic epithelial damage *in vivo* by reducing DNA damage, a pivotal early-stage event in CRC pathogenesis.

## Contributions

C.I.R.G., G.McD., R.L., I.R. and A.C. were involved in study design; C.I.R.G, G.O’C in study conduct. S.D., C.L., G.McD., G.P.-C., J.M.M.-R., K.M.T, T.M.A. C.I.R.G., and J.W.A. were involved in experimental and data analysis. The manuscript was prepared by S.D., G.P.-C., N.G.T., L.K.P., D.D.R., C.L., I.R., G.McD., A.C. and C.I.R.G with contributions from all the authors.

## Declaration of competing interest

The authors declare no conflicts of interest.
