# Supplementary material for: Ex vivo fecal fermentation of human ileal fluid collected after raspberry consumption modifies (poly)phenolics and modulates genoprotective effects in colonic epithelial cells
Source: Redox Biol. 2021 Jan 12;40:101862. doi: 10.1016/j.redox.2021.101862 (PMC7823050; doi:10.1016/j.redox.2021.101862)
Supplement: Multimedia component 1 [file mmc1.docx]

**Supplementary Information**

Ex vivo fecal fermentation of human ileal fluid collected after raspberry consumption modifies (poly)phenolics and modulates genoprotective effects in colonic epithelial cells.

Sara Dobani^a^, Cheryl Latimer^a^, Gordon J. McDougall^b^, J. William Allwood^b^, Gema Pereira-Caro^c^, José Manuel Moreno-Rojas^c^, Nigel G. Ternan^a^, L. Kirsty Pourshahidi^a^, Roger Lawther^d^, Kieran M. Tuohy^e^, Daniele Del Rio^f^, Gloria O’Connor^d^, Ian Rowland^g^, Tahani Mazyad Almutairi^h^, Alan Crozier^h,i^, Chris I. R. Gill^a^

*^a^Nutrition Innovation Centre for Food and Health, University of Ulster, Coleraine, Northern Ireland, UK*

*^b^Environmental and Biochemical Sciences Department, The James Hutton Institute, Invergowrie, Dundee, Scotland, UK*

*^c^Department of Food Science and Health, IFAPA-Alameda del Obispo, SN, Córdoba, Spain*

*^d^Altnagelvin Area Hospital, Londonderry, Northern Ireland, UK*

*^e^Food Quality and Nutrition Department, Fondazione Edmund Mach, San Michele all'Adige, Italy*

*^f^Department of Veterinary Science, University of Parma, Parma, Italy*

*^g^Department of Food and Nutritional Sciences, University of Reading, Reading, UK*

*^h^Department of Chemistry, King Saud University, Riyadh, Saudi Arabia*

*^i^Department of Nutrition University of California, Davis, California, USA*

**Table S1**. HPLC-HRMS characteristics of polyphenols presented in ileal fluid collected 0-8 h after 300 g of raspberry intake. Chromatographic conditions were as follows: reverse phase separation was carried out using a 100 x 2.1 mm i.d. 1.8 µm Zorbax SB C18 (Agilent) maintained at 40 °C and eluted at a flow rate of 0.2 mL/min with a 50 min gradient of 3-70% of 0.1% acidic methanol in 0.1% aqueous formic acid.

| **Compound** | **RT (min)** | **Chemical formula** | **[*m/z*]+^a^** | **MSI MI**  **level^c^** |
| --- | --- | --- | --- | --- |
| Cyanidin-3,5-*O*-diglucoside | 18.1 | C_27_H_30_O_16_ | 611.1606 | 2 |
| Cyanidin-3-*O*-sophoroside | 18.9 | C_27_H_30_O_16_ | 611.1606 | 2 |
| Cyanidin-3-*O*-(2"-*O*-glucosyl)rutinoside | 19.9 | C_33_H_40_O_20_ | 757.2185 | 2 |
| Pelargonidin-3-*O*-sophoroside | 23.5 | C_27_H_30_O_15_ | 595.1657 | 2 |
| Cyanidin-3-*O*-glucoside | 24.2 | C_21_H_20_O_11_ | 449.1078 | 1 |
| Cyanidin-3-*O*-(2"-*O*-xylosyl)rutinoside | 25.2 | C_32_H_38_O_19_ | 727.2080 | 2 |
| Cyanidin-3-*O*- rutinoside | 27.1 | C_27_H_30_O_15_ | 594.1579 | 2 |
| Pelargonidin-3-*O*-glucoside | 28.9 | C_21_H_20_O_10_ | 433.11292 | 1 |
| Ellagic acid-*O*-pentoside | 34.6 | C_19_H_14_O_12_ ^b^ | 433.0401 | 2 |
| Ellagic acid | 35.6 | C_14_H_6_O_8_ ^b^ | 300.9978 | 1 |
| Sanguiin H-10 | 18.9 | C_68_H_48_O_44_^b^ | 1567.14347 | 2 |
| Sanguiin H-6 | 26.0 | C_82_H_54_O_52_^b^ | 1869.1497 | 2 |
| Lambertianin C | 29.2 | C_123_H_80_O_78_ ^b^ | 2803.2209 | 2 |

^a^ Chemical formula [*m/z*]^+^ indicated the protonated molecule [M-H]^+^

^b^Chemical formula [*m/z*]^-^ indicated the deprotonated molecule [M-H]^-^

^c^ Metabolite Standards Initiative metabolite identification (MSIMI) levels [1]. Reference compounds were available for all compounds identified at MSISI level 1.

**Table S2.** GC retention times and characteristic MS ions of trimethylsilyl derivatives of phenolic and aromatic acid catabolites in ileal fluid collected 0-8 h after 300 g of raspberry intake. Commercially available standard compounds were used for all the phenolic acids quantified. The GC conditions were previously described by Pereira-Caro et al. [2].

| **Phenolic catabolites** | **Rt (min)** | **Target ion (*m/z*)** | **Qualifier ions (*m/z*)** |
| --- | --- | --- | --- |
| Benzoic acid | 5.8 | 105 | 179, 135, 77 |
| Benzene-1,2-diol (catechol) | 6.4 | 254 | 73, 239, 151 |
| 3-(Phenyl)propionic acid | 7.4 | 104 | 75, 222 |
| Benzene 1,2,3-triol (pyrogallol) | 8.8 | 239 | 73, 342 |
| 2-Hydroxy-3-(phenyl)propanoic acid [3-(phenyl)lactic acid] | 9.5 | 193 | 73, 147 |
| 3′-Hydroxyphenylacetic acid | 10.5 | 267 | 73, 193, 282 |
| 4-Hydroxybenzoic acid | 10.9 | 267 | 282, 223, 193, 73 |
| 4′-Hydroxyphenylacetic acid | 11.1 | 73 | 296, 281, 179, 164 |
| 3-(3´-Hydroxyphenyl)propionic acid | 13.3 | 205 | 192 ,310, 177, 73 |
| 3-(4´-Hydroxyphenyl)propionic acid | 14.3 | 179 | 192, 73, 310 |
| 3,4-Dihydroxybenzoic acid | 15.4 | 193 | 73, 370, 355 |
| 3-(3´,4´-Dihydroxyphenyl)propionic acid | 20.6 | 73 | 179, 267, 398, 383 |
| 3-(3′-Hydroxy-4′-methoxyphenyl)propionic acid | 18.3 | 340 | 209, 192, 179, 73 |
| 2-Hydroxy-3-(4′-hydroxyphenyl)propanoic acid [3-(4′-hydroxyphenyl)lactic acid] | 18.9 | 179 | 73, 147, 308 |
| 3′,4′-Dihydroxycinnamic acid [caffeic acid] | 22.1 | 308 | 73, 323, 249 |
| 4′-Hydroxycinnamic acid [coumaric acid] | 19.8 | 219 | 73, 249, 293, 308 |
| 3′-Hydroxy-4-methoxycinnamic acid | 27.1 | 338 | 73, 323, 249, 308 |

**Table S3.** Sequences of the forward (F, 5’ -> 3’) and reverse (R, 3’ -> 5’) primers designed for the Nrf2, HO-1, NQO1, HPRT, β-actin genes. The amplicon length is expressed as base pairs (bp).

| **Gene** | **Full Name** | **Primer sequence** | **Amplicon Length (bp)** |
| --- | --- | --- | --- |
| Nrf2 | Nuclear factor (erythroid derived 2)-like 2 | F: AAACCAGTGGATCTGCCAAC  R: GCAATGAAGACTGGGCTCTC | 190 |
| HO-1 | Heme oxygenase 1 | F: ATGACACCAAGGACCAGAGC  R: GTGTAAGGACCCATCGGAGA | 153 |
| NQ01 | NAD(P)H dehydrogenase, quinone 1 | F: AGGACCCTTCCGGAGTAAGA  R: AGGCTGCTTGGAGCAAAATA | 279 |
| HPRT | Hypoxanthine phosphoribosyl transferase | F: AGCTTGCGACCTTGACCAT  R: GACCAGTCAACAGGGGACAT | 166 |
| β-actin | Beta actin | F: GGACTTCGAGCAAGAGATGG  R: AGCACTGTGTTGGCGTAAG | 234 |

**Table S4**. Quantities of anthocyanins, ellagic acid derivatives and ellagitannins present in 300 g of a raspberry smoothy. Data expressed as µmol ± SDM (n=3).

| **Compound** | **µmol/300 g ± SDM** |
| --- | --- |
| Cyanidin-3,5-*O*-diglucoside | 0.6 ± 0.1 |
| Cyanidin-3-*O*-sophoroside | 22 ± 1 |
| Cyanidin-3-*O*-(2"-*O*-glucosyl)rutinoside | 4.2 ± 0.3 |
| Pelargonidin-3-*O*-sophoroside | 0.3 ± 0.0 |
| Cyanidin-3-*O*-glucoside | 9 ± 1 |
| Cyanidin-3-*O*-(2"-*O*-xylosyl)rutinoside | 0.3 ± 0.1 |
| Cyanidin-3-*O*-rutinoside | 2.2 |
| Pelargonidin-3-*O*-glucoside | 0.1 ± 0.1 |
| **Total anthocyanins** | **39 ± 3** |
| Ellagic acid-*O*-pentoside | 1.2 ± 0.1 |
| Ellagic acid | 4.8 ± 0.1 |
| **Total ellagic acid** | **6.0 ± 0.2** |
| Sanguiin H-10 | 43 ± 3 |
| Sanguiin H-6 | 61 ± 3 |
| Lambertianin C | 20 ± 2 |
| **Total ellagitannins** | **124 ± 8** |

**Table S5.** The quantities of phenolic acids and aromatic catabolites present in ileal fluid from subjects ,who consumed 300 g of raspberry, before (0 h) and after (24 h) fermentation with a human fecal sample. Data expressed as mean values in µmol ± SDM (n=3).

|  | S1-0 h | S1-24 h | S2-0 h | S2-24 h | S3-0 h | S3-24 h | S4-0 h | S4-24 h | S5-0 h | S5-24 h | S6-0 h | S6-24 h |
| --- | --- | --- | --- | --- | --- | --- | --- | --- | --- | --- | --- | --- |
| *Cinnamic acid derivatives* |  |  |  |  |  |  |  |  |  |  |  |  |
| 4′-Hydroxycinnamic acid | n.d. | 2.1 ± 0.1 | 3.1 ± 0.7 | n.d. | 1.2 ± 0.3 | 0.6 ± 0.1 | n.d. | n.d. | n.d. | n.d. | n.d. | n.d. |
| 3´,4´-Dihydroxycinnamic acid | n.d. | 40 ± 5 | 25 ± 6 | n.d. | 2.1 ± 0.3 | n.d. | n.d. | n.d. | n.d. | n.d. | n.d. | n.d. |
| 3’-Hydroxy-4’-methoxycinnamic acid | n.d. | n.d. | n.d. | n.d. | 1.3 ± 0.2 | n.d. | n.d. | n.d. | n.d. | n.d. | n.d. | n.d. |
| *Phenylpropanoic acid derivatives* |  |  |  |  |  |  |  |  |  |  |  |  |
| 3-(3′-Hydroxyphenyl)propanoic acid | n.d. | n.d. | n.d. | n.d. | 2.4 ± 0.3 | 4.1 ± 0.6 | 1.7 ± 0.2 | 2.1 ± 0.8 | 8 ± 1 | 7.2 ± 0.2 | n.d. | n.d. |
| 3-(4′-Hydroxyphenyl)propanoic acid | n.d. | 2.2 ± 0.8 | 0.2 ± 0.1 | 0.2 ± 0.1 | n.d. | n.d. | n.d. | 2.6 ± 0.2 | n.d. | n.d. | n.d. | n.d. |
| 3-(3′,4′-Dihydroxyphenyl)propanoic acid | n.d. | n.d. | n.d. | n.d. | n.d. | n.d. | 1.0 ± 0.2 | n.d. | n.d. | n.d. | 7.6 ± 0.5 | n.d. |
| 3-(4′-Hydroxy-3′-methoxyphenyl)propanoic acid | n.d. | 11 ± 1 | 9 ± 2 | n.d. | n.d. | n.d. | n.d. | n.d. | n.d. | n.d. | 13 ± 2 | 4.0 ± 0.6 |
| 3-(Phenyl)propanoic acid | n.d. | 4.4 ± 0.6 | 7 ± 1 | 1.0 ± 0.7 | 1.5 ± 0.2 | 1.2 ± 0.2 | 0.8 ± 0.2 | 3.1 ± 1.7 | 1.6 ± 0.4 | 1.1 ± 0.1 | 0.8 ± 0.3 | 2.9 ± 0.7 |
| 2-Hydroxy-3-(4′-hydroxyphenyl)propanoic acid | n.d. | n.d. | n.d. | n.d. | 0.3 ± 0.1 | n.d. | 1.2 ± 0.2 | n.d. | 5.3 ± 0.2 | n.d. | 1.7 ± 0.7 | n.d. |
| 2-Hydroxy-3-(phenyl)propanoic acid | 3.1 ± 0.3 | 3.6 ± 0.6 | 1.3 ± 0.8 | 2 ± 1 | 5 ± 1 | 0.4 ± 0.1 | 3.9 ± 1.0 | n.d. | n.d. | n.d. | 4.5 ± 0.3 | 13 ± 8 |
| *Phenylacetic acid derivatives* |  |  |  |  |  |  |  |  |  |  |  |  |
| 4′-Hydroxyphenylacetic acid | 90 ± 12 | 104 ± 16 | 40 ± 8 | 4 ± 1 | 1.0 ± 0.2 | 1.3 ± 0.2 | 3.1 ± 0.3 | n.d. | 57 ± 2 | 3.2 ± 0.2 | 2.4 ± 0.4 | 21 ± 3 |
| 3′-Hydroxyphenylacetic acid | 40 ± 5 | 53 ± 6 | 15 ± 4 | 1.8 ± 0.3 | 0.3 ± 0.2 | 1.2 ± 0.2 | n.d. | n.d. | n.d. | n.d. | n.d. | n.d. |
| *Benzoic acids* |  |  |  |  |  |  |  |  |  |  |  |  |
| 3,4-Dihydroxybenzoic acid | n.d. | 16 ± 1 | 6.7 ± 0.2 | 2.1 ± 0.3 | n.d. | n.d. | n.d. | 0.8 ± 0.4 | n.d. | n.d. | n.d. | 16 ± 2 |
| 4-Hydroxybenzoic acid | 24 ± 7 | 9.2 ± 5 | 6 ± 2 | 7.2 ± 0.9 | 0.4 ± 0.1 | 10 ± 1 | 4 ± 1 | 8.1 ± 0.7 | n.d. | n.d. | 12 ± 3 | 14 ± 3 |
| Benzoic acid | 56 ± 1 | 98 ± 10 | 16 ± 6 | 18 ± 1 | 31 ± 1 | 18 ± 1 | 24 ± 2 | 22 ± 2 | 18 ± 1 | 13 ± 4 | 12 ± 2 | 34 ±11 |
| *Benzenetriols* |  |  |  |  |  |  |  |  |  |  |  |  |
| Benzene-1,2-diol (catechol) | 474 ± 58 | 718 ± 7 | 522 ± 61 | 905 ± 23 | 44 ± 1 | 102 ± 8 | 151 ± 3 | 384 ± 16 | 249 ± 82 | 61 ± 2 | 50 ± 5 | 445 ± 45 |
| Benzene-1,2,3-triol (pyrogallol) | 3.0 ± 0.3 | 3.0 ± 0.4 | 1.3 ± 0.3 | 3.7 ± 0.3 | 1.0 ± 0.2 | 0.8 ± 0.2 | 1.2 ± 0.9 | 0.5 ± 0.2 | 4.2 ± 0.7 | 2 ± 1 | n.d. | n.d. |
| **Total** | **690 ± 84** | **1064 ± 59** | **653 ± 92** | **945 ± 29** | **91 ± 5** | **140 ± 12** | **192 ± 9** | **423 ± 22** | **343 ± 87** | **87 ± 7** | **104 ± 14** | **550 ± 73** |

**Table S5.** Continued

|  | S8-0 h | S8-24 h | S9-0 h | S9-24 h | S10-0 h | S10-24 h | S11-0 h | S11-24 h | S12-0 h | S12-24 h |
| --- | --- | --- | --- | --- | --- | --- | --- | --- | --- | --- |
| *Cinnamic acid derivatives* |  |  |  |  |  |  |  |  |  |  |
| 4′-Hydroxycinnamic acid | 0.4 ± 0.1 | n.d. | n.d. | n.d. | n.d. | n.d. | n.d. | 8.9 ± 0.3 | n.d. | n.d. |
| 3´,4´-Dihydroxycinnamic acid | n.d. | n.d. | n.d. | n.d. | n.d. | n.d. | n.d. | n.d. | n.d. | n.d. |
| 3’-Hydroxy-4’-methoxycinnamic acid | n.d. | n.d. | n.d. | n.d. | n.d. | 2.3 ± 0.8 | n.d. | 9.5 ± 0.6 | 6.5 ± 0.4 | n.d. |
| *Phenylpropanoic acid derivatives* |  |  |  |  |  |  |  |  |  |  |
| 3-(3′-Hydroxyphenyl)propanoic acid | 1.1 ± 0.2 | 1.9 ± 0.6 | n.d. | n.d. | n.d. | n.d. | n.d. | n.d. | n.d. | n.d. |
| 3-(4′-Hydroxyphenyl)propanoic acid | n.d. | n.d. | n.d. | n.d. | 2.8 ± 0.5 | n.d. | n.d. | 0.2 ± 0.1 | n.d. | n.d. |
| 3-(3′,4′-Dihydroxyphenyl)propanoic acid | 1.3 ± 0.2 | n.d. | n.d. | 0.7 ± 0.2 | n.d. | 0.1 ± 0.1 | n.d. | 17 ± 1 | n.d. | n.d. |
| 3-(4′-Hydroxy-3′-methoxyphenyl)propanoic acid | n.d. | n.d. | n.d. | n.d. | n.d. | 2.7 ± 0.2 | 6 ± 1 | 13 ± 2 | n.d. | 2.4 ± 0.2 |
| 3-(Phenyl)propanoic acid | 0.3 ± 0.1 | 1.1 ± 0.4 | 0.3 ± 0.1 | 0.1 ± 0.1 | 3.8 ± 0.9 | 0.5 ± 0.3 | 2.7 ± 0.7 | 19 ± 1 | 6 ± 1 | 0.1 ± 0.1 |
| 2-Hydroxy-3-(4′-hydroxyphenyl)propanoic acid | 0.4 ± 0.1 | n.d. | n.d. | n.d. | n.d. | n.d. | 3.1 ± 0.2 | n.d. | 1.2 ± 0.3 | n.d. |
| 2-Hydroxy-3-(phenyl)propanoic acid | 2.3 ± 0.5 | n.d. | 1.2 ± 0.2 | n.d. | 1.8 ± 0.6 | n.d. | 2.7 ± 0.1 | 11 ± 1 | 0.4 ± 0.1 | n.d. |
| *Phenylacetic acid derivatives* |  |  |  |  |  |  |  |  |  |  |
| 4′-Hydroxyphenylacetic acid | 2.8 ± 0.5 | n.d. | n.d. | n.d. | 26 ± 4 | 51 ± 10 | 8.5 ± 0.3 | 15 ± 1 | 7 ± 1 | 0.3 ± 0.1 |
| 3′-Hydroxyphenylacetic acid | n.d. | n.d. | n.d. | n.d. | n.d. | 26 ± 6 | n.d. | n.d. | n.d. | n.d. |
| *Benzoic acids* |  |  |  |  |  |  |  |  |  |  |
| 3,4-Dihydroxybenzoic acid | n.d. | 3.9 ± 0.8 | n.d. | 0.8 ± 0.2 | n.d. | 21 ± 7 | n.d. | 33 ± 1 | n.d. | 6.2 ± 0.6 |
| 4-Hydroxybenzoic acid | n.d. | n.d. | n.d. | 3.8 ± 0.4 | 0.5 ± 0.1 | 1.6 ± 0.7 | n.d. | 26 ± 3 | 3 ± 1 | n.d. |
| Benzoic acid | 36 ± 13 | 19 ± 12 | 4.7 ± 0.3 | 37 ± 2 | 30 ± 3 | 132 ± 5 | 44 ± 6 | 12 ± 6 | 18 ± 2 | 24 ± 7 |
| *Benzenetriols* |  |  |  |  |  |  |  |  |  |  |
| Benzene-1,2-diol (catechol) | 12 ± 5 | 11 ± 3.6 | 69 ± 11 | 84 ± 10 | 111 ± 14 | 368 ± 48 | 34 ± 2 | 120 ± 16 | 23 ± 1 | 140 ± 5 |
| Benzene-1,2,3-triol (pyrogallol) | 1.6 ± 0.9 | 2.7 ± 0.7 | 2.7 ± 1.8 | 0.1 ± 0.1 | 0.8 ± 0.2 | 1.5 ± 0.7 | 0.4 ± 0.3 | 0.4 ± 1.8 | 0.1 ± 0.1 | 0.2 ± 0.1 |
| **Total** | **58 ± 21** | **40 ± 18** | **78 ± 13** | **126 ± 13** | **177 ± 23** | **607 ± 79** | **101 ± 11** | **285 ± 36** | **65 ± 6** | **173 ± 13** |

**Table S6.** A summary of the impact of fecal fermentation on the concentration of individual phenolic acids and aromatic catabolites present in ileal fluid from 11 subjects who consumed 300 g of raspberries before (0 h) and after (24 h) fermentation with human fecal samples. Data expressed as µM ± SEM (n=3). The mean value post-fermentation is statistically different compared with the mean value pre-fermentation. One-way ANOVA and Dunnet T test, (*)p< 0.05.

|  | **0 h**  **Range** | **24 h**  **Range** | **0 h**  **Mean ± SEM** | **24 h**  **Mean ± SEM** |
| --- | --- | --- | --- | --- |
| **Cinnamic acid derivatives** |  |  |  |  |
| 4′-Hydroxycinnamic acid | n.d. -14.6 | n.d. - 4 | 1.8 ± 0.4 | 0.6 ± 0.1* |
| 3´,4´-Dihydroxycinnamic acid | n.d. - 51 | n.d - 12 | 8.1 ± 1.7 | 1.1 ± 0.3* |
| 3’-Hydroxy-4’-methoxycinnamic acid | n.d. – 17 | n.d. - 13 | 1.8 ± 0.5 | 1.8 ± 0.4 |
| **Phenylpropanoic acid derivatives** |  |  |  |  |
| 3-(3′-Hydroxyphenyl)propanoic acid | n.d. - 16 | n.d. – 84 | 3.8 ± 0.5 | 18.5 ± 2.7* |
| 3-(4′-Hydroxyphenyl)propanoic acid | n.d. - 51 | n.d. - 26 | 6.1 ± 1.4 | 7.4 ± 0.9 |
| 3-(3′,4′-Dihydroxyphenyl)propanoic acid | n.d. - 27 | n.d. - 6 | 4.0 ± 0.7 | 0.8 ± 0.2* |
| 3-(4′-Hydroxy-3′-methoxyphenyl)propanoic acid | n.d. – 38 | n.d.- 18.4 | 4.2 ± 1.0 | 3.4 ± 0.6 |
| 3-(Phenyl)propanoic acid | n.d. - 33 | n.d.- 144 | 6.0 ± 0.9 | 20 ± 4* |
| 2-Hydroxy-3-(4′-hydroxyphenyl)propanoic acid | n.d. | n.d.-39 | n.d. | 10 ± 1* |
| 2-Hydroxy-3-(phenyl)propanoic acid | n.d. - 24 | n.d. - 137 | 5.7 ± 0.8 | 26 ± 3* |
| **Phenylacetic acid derivatives** |  |  |  |  |
| 4′-Hydroxyphenylacetic acid | n.d. - 191 | n.d. - 43 | 33 ± 5 | 8 ± 1* |
| 3′-Hydroxyphenylacetic acid | n.d. - 86 | n.d. - 12 | 11.0 ± 2.4 | 2.1 ± 0.4* |
| **Benzoic acids** |  |  |  |  |
| 3,4-Dihydroxybenzoic acid | n.d. - 33 | n.d. - 6 | 12 ± 1 | 1.7 ± 0.2* |
| 4-Hydroxybenzoic acid | n.d. - 87 | 3.6 - 51 | 19 ± 3 | 21 ± 2 |
| Benzoic acid | 14 - 246 | 30 - 357 | 76 ± 7 | 149 ± 8* |
| **Benzenetriols** |  |  |  |  |
| Benzene-1,2-diol (catechol) | 3.6 - 1564 | n.d. - 1600 | 386 ± 60 | 533 ± 145* |
| Benzene-1,2,3-triol (pyrogallol) | n.d. - 10 | n.d. - 8 | 3.7 ± 1.0 | 3.1 ± 0.2 |
| **Total phenolics** | **54 - 1661** | **162 - 1962** | **581 ± 51** | **807± 56*** |

**REFERENCES**

[1] L.W. Summer, A. Amberg, D. Barrett, M.H. Beale, R. Beger, C.A. Daykin, T.W. Fan, O. Fiehn, R. Goodacre, J.L. Griffin, et al. Proposed minimum reporting standards for chemical analysis. Chemical Analysis Working Group (CAWG) Metabolomics Standards Initiative (MSI). Metabolomics 3 (2007) 211-221.

[2] G, Pereira-Caro, G. Borges, J. van der Hooft, M.N. Clifford, D. Del Rio, M.E. Lean, S.A. Roberts, M.B. Kellerhals, A. Crozier. Orange juice (poly)phenols are highly bioavailable in humans. Am. J. Clin. Nutr. 100 (2014) 1378–1384.
